# Osteolysis After HINTEGRA Total Ankle Replacement: Radiographic Patterns, Alignment Associations, and Long-Term Outcomes

**DOI:** 10.1177/24730114251363495

**Published:** 2025-08-29

**Authors:** Eric Locke, Roxane Heroux-Legault, Maram Alothman, Zaid Jibri, Brad Meulenkamp, Karl-André Lalonde

**Affiliations:** 1 Division of Orthopaedic Surgery, University of Ottawa, Ottawa, ON, Canada; 2 Department of Radiology, University of Ottawa, Ottawa, ON, Canada; 3 King Faisal Specialist Hospital and Research Center in Riyadh, Saudi Arabia; 4 Greater Niagara Medical Imaging (GNMI), Toronto

**Keywords:** HINTEGRA, total ankle replacement, osteolysis

## Abstract

**Background:**

Total ankle replacement (TAR) is a surgical option for patients with ankle arthritis who have failed conservative measures. Newer implants have markedly improved; however, osteolysis causing aseptic loosening continues to be a main cause of TAR failure. The objective of this study was to review the HINTEGRA TAR experience at a single institution specifically evaluating the presence and outcomes of osteolysis.

**Methods:**

Retrospective study including all HINTEGRA TARs completed by 1 experienced foot and ankle surgeon from 2006 to 2014. Radiographs were reviewed, assessing for implant positioning, presence, location, and progression of cysts as well as relationship between osteolysis with reoperations and revisions.

**Results:**

Fifty-one TARs were identified with radiographic follow-up of 5.8 ± 3.5 years. Eighty-four cysts were detected in 37 patients, with increasing number and size of cysts being correlated to length of time from surgery. The most common location was the posterior tibia. Thirteen patients had enlarging cysts identified over time, with the lateral malleolus being the most common location. Seven patients met criteria for malaligned prosthesis, 12 patients required a reoperation, and 2 patients experienced implant failure.

**Conclusion:**

Osteolysis is a very common finding after TAR using the HINTEGRA prosthesis, specifically on long-term radiographic follow-up. Progressive cysts and prosthesis coronal malalignment appear to be risk factors for developing osteolysis, prosthesis loosening, and reoperation. Most cysts did not result in clinical failure, but progressive lesions identified beyond 1 year warrant closer monitoring. This study also shows excellent and reliable outcomes of the HINTEGRA TAR compared with designer surgeons with acceptable complication and revision rates.

**Level of Evidence:**

Level IV, Case series

## Graphical abstract

**Graphical Abstract:**
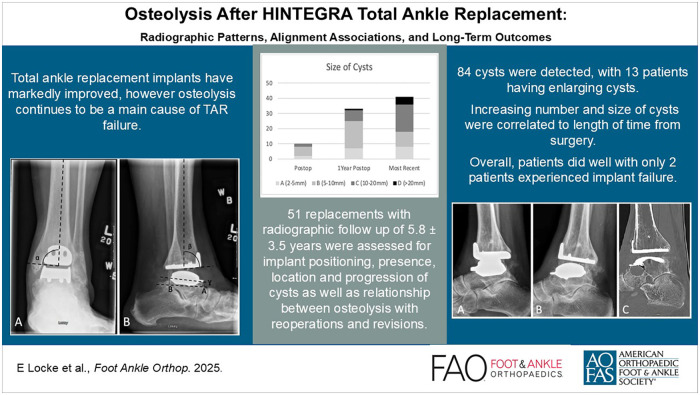

## Introduction

Ankle arthritis is estimated to affect 1% of the world’s adult population, with posttraumatic ankle arthritis being the most common etiology.^15,31^ End-stage ankle arthritis can cause substantial functional impairment and decreased quality of life.^28^ Total ankle replacement (TAR) is a surgical option, preferred by patients^8^ who have failed conservative measures with several advantages to arthrodesis.^6,25,27,32^ Despite cautious patient selection,^4^ failure rates of TAR remain higher than other total joint arthroplasties.^13,24^ However, survivorship of third-generation implants has been markedly improved compared with previous generations,^3^ with 10-year implant survival rates varying between 70% and 90%.^2,13,22,36,37^

Osteolysis is believed to be an inflammatory reaction to wear particles produced by shear stress on the polyethylene bearing surface.^1^ In regard to TARs, anatomical, structural, vascular, and mechanical factors together with implant design appear to explain the osteolytic changes in periimplant tissues.^23^ It is a known and recognized complication of TAR associated with aseptic loosening, the most common reason for TAR revision^11,25^ and failure.^22,34^ However, the presence of osteolysis varies immensely in the literature from 0% to 86%,^14^ with proposed types of osteolysis emerging^21^ and links to prosthesis malalignment causing increased peak and contact pressures.^11,12^

The HINTEGRA prosthesis is a mobile-bearing, 3-component TAR system.^19^ The midterm survivorship of the HINTEGRA prosthesis is comparable with that of other third-generation TAR with varying survival rates between 82% and 94% at 5 years^3,7,20^ and 84% at 10 years.^3^ Osteolysis has been identified in 37.4%^34^ and 48%^9^ of studies using HINTEGRA. More recent research suggests fewer implant failures with the HINTEGRA prosthesis, along with fewer patients receiving additional surgery for implant loosening^37^ compared with other prosthesis designs. The objective of this study was to review the HINTEGRA TAR experience at a single institution over an 8-year period between 2006 and 2014, specifically evaluating the radiographic formation, location, and progression of cysts and the relation between cysts and prosthesis alignment, reoperations, and implant survivorship.

## Methods

### Study Design

This retrospective study included all TARs done at The Ottawa Hospital (TOH) from January 1, 2006, to December 31, 2014, using the HINTEGRA prosthesis. The protocol was approved by the Ottawa Health Science Network Research Ethics Board. All TARs were performed by a senior staff foot and ankle surgeon (K.L.). The surgeon had TAR experience since 2004, using a variety of implants including HINTEGRA over this time period.

### Participants

Cases were identified through a Data Warehouse search at TOH, including all operations involving a TAR. Charts were analyzed for patient demographics, surgical indication, relevant prior surgeries, operative procedure and prosthesis, surgical complications, as well as need for reoperation. All patients who underwent a TAR using the HINTEGRA prosthesis between 2006 and 2014 were included. Any incorrect prosthesis or procedure such as ankle fusion were excluded. Weightbearing anterior-posterior (AP) and lateral preoperative, postoperative, 1-year postoperative, and long-term weightbearing radiographs were reviewed. Patients with less than 5 years of radiographic of follow-up were contacted via phone to assess prosthesis survivorship.

### Implant and Operative Technique

The HINTEGRA ankle prosthesis is a nonconstrained, 3-component system. The HINTEGRA prosthesis uses all available bone surface for support with minimized contact stress between articulating surfaces. A porous-coated surface covered by porous titanium and hydroxyapatite (HA) allows for early fixation of the implant through HA bonding and later fixation by secure ingrowth of the bone within beaded interstices.^19^

A direct anterior approach to the ankle was used in all cases. The tibial cut was performed using the alignment guide and fluoroscopic control, with specific attention to protecting the posteromedial structures. The talus was then prepared and cut using the talar cutting guide. The tibia was then sized, and trial components were tested. Once adequate fit and ligamentous balance was achieved, the trial components were removed, and definitive components were inserted and impacted after irrigation of the wound. An appropriate-size polyethylene bearing was selected, and intraoperative range of motion was assessed. At this point, additional procedures such as Achilles lengthening, lateral ligament reconstruction, calcaneal osteotomies or mid- and forefoot corrections to achieve ideal osseous and ligamentous balance were performed whenever necessary. The wounds were then irrigated, and layered closure was performed in typical fashion.

### Radiographic Cysts

**Figure 1.**
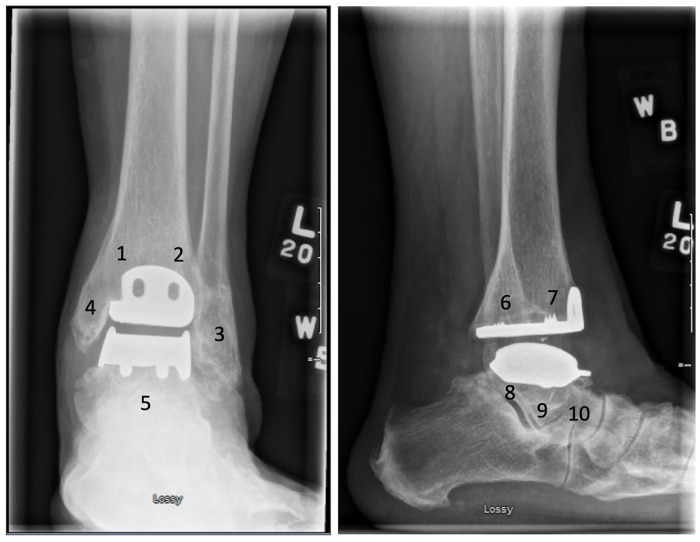
Besse et al^5^ (2009) classification for location of cysts.

Evaluation of the presence, location, and progression of cysts was performed by a fellowship-trained MSK radiologist, who assessed weightbearing anteroposterior (AP) and lateral preoperative, postoperative, 1-year postoperative, and long-term radiographs. Postoperative weightbearing films were typically obtained at an average of 6 weeks postoperatively, which will be considered as the postoperative films throughout the study. Cysts that were present preoperatively were not included. If patients required a reoperation or revision, their last radiographs prior to surgery were considered their most recent imaging. In cases where reoperation did not involve concern for osteolysis or prothesis failure, their most recent imaging was their true last follow-up, despite the subsequent surgery. Radiographs were divided into 5 zones on the AP view and 5 zones on the lateral view using the classification proposed by Besse et al^5^ (Figure 1). Any new radiolucency greater than 2 mm was considered a cyst. The size of each cyst was determined using McKesson radiology PACS station measured to the nearest 0.1 mm and was then classified into one of 4 size categories: 2 to 5 mm, 5 to 10 mm, 10 to 20 mm, and greater than 20 mm. Progression of a cyst was defined as an enlarging cyst that required recategorization due to new size.

### Radiographic Alignment

**Figure 2.**
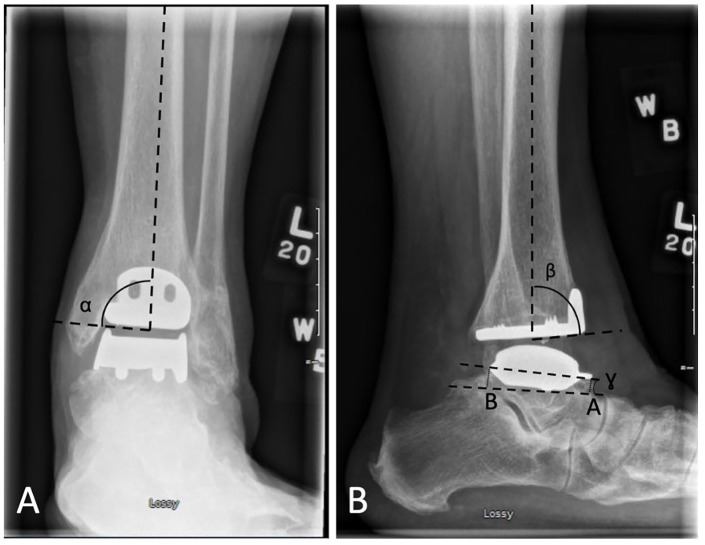
Radiographic alignment measurements (A) alpha angle, (B) beta angle, gamma angle, and A and B distances.

Radiographic alignment evaluation was performed independently by 2 reviewers, an orthopaedic resident and orthopaedic foot and ankle fellow, who assessed weightbearing AP and lateral postoperative, 1-year postoperative, and long-term weightbearing radiographs. Alignment was measured to the nearest 0.1 degrees using McKesson radiology PACS station, with angular and distance values measured digitally (Figure 2). The alpha angle is the angle on the AP view, between the longitudinal axis of the tibia and the articulating surface of the tibial component; beta angle is the angle on the lateral view, between the longitudinal axis of the tibia and the articulating surface of the tibial component; gamma angle is the angle on the lateral view, between a line drawn through the anterior shield and the posterior edge of the talar component and a line drawn between the dorsal aspect of the talonavicular joint and the calcaneal tubercle. The longitudinal axis of the tibia was determined using 2 points in the middle of the medullary canal on both the AP and lateral radiographs. The alpha and beta angles were then converted in relation to the longitudinal axis of the tibia, where a positive alpha angle indicates valgus, a negative alpha angle indicates varus, a positive beta indicates anterior opening, and a negative beta indicates posterior opening. The “A distance” is the perpendicular distance on the lateral view, from the most anterior part of the talar component to a line drawn between the dorsal aspect of the talonavicular joint and the calcaneal tubercle; and “B distance” the perpendicular distance on the lateral view, from the most posterior part of the talar component to the same line as described under A. All measurements with a >2 degrees difference between the 2 evaluators were reviewed and remeasured to resolve any inconsistencies by consensus.

Initial prosthesis alignment was assessed for malalignment and defined as placement of the tibial component with an angular deviation greater than 5 degrees or talar component in inappropriate fixation.^19^ Loosening of components will be kept consistent with previous literature defining tibial loosening as a change in position greater than 2 degrees of the flat base of the component in relation to the long axis of the tibia in the AP or lateral view. Loosening of the talar component will be defined as subsidence into the talar bone greater than 5 mm, or change in the position greater than 5 degrees relative to the line drawn from the top of the talonavicular joint to the tuberosity of the calcaneus on the lateral radiograph.^3,19,34^

### Implant Survivorship and Reoperations

Chart review was performed to determine length of follow-up and implant survivorship, and identify any surgical complications. Length of radiographic follow-up was determined for all patients by the time of their last radiographs from date of surgery. Thirteen patients with less than 5 years of radiographic follow-up were contacted via phone to assess for any missed reoperations or revision not performed at TOH. Implant survivorship was calculated based on date of surgery to either last date of radiographic follow-up, date of contact via phone, or revision surgery date. Implant failure was determined using previous definition^18^ as patients who required removal or exchange of one of the components. Reoperations were classified using the Canadian Orthopaedic Foot and Ankle Surgery’s Coding System for Reoperations Following Total Ankle Replacement and Ankle Arthrodesis, otherwise known as CROCS.^35^

### Data Analysis

Alignment of prosthesis (alpha, beta, gamma, A and B) were reported descriptively using mean and SD. Change in alignment from postoperative to 1 year and to most recent available measurement were calculated and reported descriptively. Frequency and proportion of cases with new cyst development at postoperative, 1 year, and most recent radiographs was reported, as well as the cumulative cyst development at 1 year and most recent radiographs. Intraclass correlation coefficients (ICCs) with 95% CIs were calculated using a mixed model. Data management and statistical analyses were performed using SAS software, version 9.4 (SAS Institute Inc, Cary, NC).

## Results

Seventy-three cases were identified for chart analysis, 22 of which were excluded (most commonly for non-HINTEGRA prosthesis or ankle fusion). Our series reviewed 51 TARs using the HINTEGRA prothesis performed on 50 patients between 2006 and 2014 (Table 1). Forty (78.4%) patients were female and the average patient age at implantation was 68 ± 8.8 years. The most common indication for surgery was posttraumatic arthritis found in 38 (75.0%) of patients. Twenty-seven patients (53.8%) had a previous procedure, 21 (40.4%) of which were open reduction internal fixation of the ankle. Average radiographic follow-up was 5.8 ± 3.5 years. Of the 13 patients contacted by phone, 9 described no subsequent surgeries to their TAR, 3 had passed away, and 1 was unreachable, extending average clinical follow-up to 8.6 years.

**Table 1.** Demographics of Patients, Including Gender, Surgical Indication, Prior Surgeries.

Characteristic	n (%) or Mean ± SD
Gender	
Male	11 (21.6)
Female	40 (78.4)
Age, y	66 ± 8.8
Radiographic follow-up, y	5.8 ± 3.5
Clinical follow-up, y	8.6 ± 3.0
Surgical indication	
Posttraumatic arthritis	38 (74.5)
Primary arthritis	5 (9.8)
Rheumatoid arthritis	2 (3.9)
Unknown	6 (11.8)
Prior surgeries	27 (52.9 )
Open reduction internal fixation	21 (41.2)
Ligament reconstruction	5 (9.8)
Subtalar arthrodesis	2 (3.9)
Debridement	1 (2.0%)

### Radiographic Cysts

**Figure 3.**
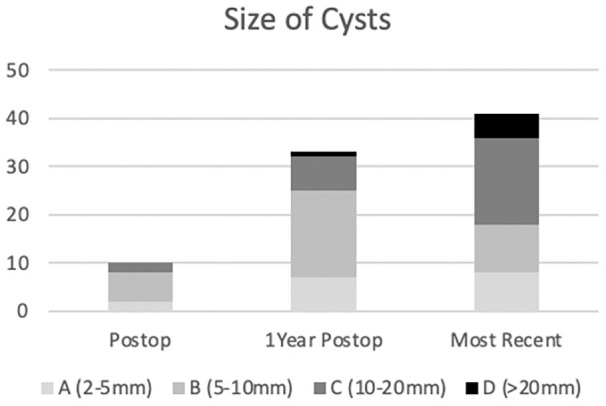
Size of new cysts.

In total, 84 cysts were detected in 37 of the 50 patients undergoing TAR. Ten patients each had a new cyst detected on postoperative radiographs, 22 patients combined for a total of 33 new cysts at 1 year postoperatively and 24 patients combined for a total of 41 new cysts on most recent radiographs. The most common cyst location was the posterior tibia or zone 6, where 17 (20.5%) cysts were identified, followed by the anterior tibia (zone 7), which contained 12 (13.6%) (Table 2). Of the 84 cysts, 17 were between 2 and 5 mm, 34 were between 5 and 10 mm, 27 were between 10 and 20 mm, and 6 were larger than 20 mm, with a trend toward larger cysts being identified further from index surgery (Figure 3).

**Table 2.** Location of New Cysts Using Besse et al^5^ (2009) Classification.

Zone	Postoperative	1-y postoperative	Most recent postoperative	Total n (%)
1	0	4	6	10 (11.9)
2	0	1	1	2 (2.4)
3	1	6	1	8 (9.5)
4	2	5	3	10 (11.9)
5	2	0	6	8 (9.5)
6	1	10	6	17 (20.2)
7	0	4	7	11 (13.1)
8	1	3	2	6 (7.1)
9	2	0	6	8 (9.5)
10	1	0	3	4 (4.8)
Total	10	33	41	84

Of the 51 patients undergoing TAR, 2 patients each had 1 cyst enlarge at 1 year postoperatively and 12 patients had 15 cysts enlarge at the most recent radiographs for a total of 13 patients and 17 progressive cysts. Furthermore, some patients had multiple progressive cysts, including 1 patient who had 3 enlarging cysts at their most recent imaging, another patient had 2 enlarging cysts at their most recent imaging, and 1 patient had an enlarging cyst at 1 year postoperatively as well as different cysts enlarging on their most recent radiographs. The most common location for cyst progression was the lateral malleolus or zone 3, where 7 (35.3%) cysts enlarged, followed by the posterior tibia or zone 6, which contained 4 (23.5%) (Table 3). Both progressive cysts that were identified on 1-year postoperative radiographs had enlarged by 1 size, as well as 11 of the 15 enlarging cysts on most recent imaging; the other 4 cysts enlarged by 2 size categories.

**Table 3.** Location of Progressing Cysts Using Besse et al^5^ (2009) Classification.

Zone	1 y Postoperative	Most Recent Postoperative	Total n (%)
1	0	0	0 (0)
2	0	0	0 (0)
3	1	5	6 (35.3)
4	0	2	2 (11.8)
5	0	3	3 (17.6)
6	0	4	4 (23.5)
7	0	1	1 (5.9)
8	0	0	0 (0)
9	0	0	0 (0)
10	1	0	1 (5.9)
Total	2	15	17

### Radiographic Alignment

Radiographic alignment for postoperative, 1-year postoperative and most recent radiographs are shown in Table 4. There were 6 patients who met definition for a malaligned prosthesis, all on the tibial side.^19^ Five were malaligned in the sagittal plane and 1 malaligned in the coronal plane. Of the 5 malaligned in the sagittal plane, 4 had identified cysts, 2 of which progressed over time, and none required any reoperation or revision. The patient who was malaligned in the coronal plane had a progressive talar cyst that fractured 6.5 years postoperatively and required subtalar fusion with bone grafting (CROCS 6).

**Table 4.** Radiographic Alignment for Postoperative, 1-Year Postoperative, and Most Recent Radiographs.

	Postoperative	1 y Postoperative	Most Recent Postoperative
Alpha, degrees	1.2 ± 1.8	1.3 ± 1.8	1.5 ± 2.1
Beta, degrees	1.6 ± 2.1	1.9 ± 1.7	2.0 ± 1.9
Gamma, degrees	8.2 ± 4.6	8.4 ± 4.4	7.8 ± 4.7
Distance A, mm	7.6 mm ± 3.1	6.8 mm ± 2.9	7.2 mm ± 2.9
Distance B, mm	13.9 mm ± 3.6	13.1 mm ± 3.5	13.1 mm ± 3.4

**Table 5.** Reoperations of Patients After TAR Based on CROCS.

CROCS	Patients	CROCS Definition
Nil		
1	39	No reoperation within or surrounding the ankle
Reoperation surrounding primary operative site		
2	1	Isolated hardware removal around the ankle
3	1	Repeat operation outside the ankle replacement, but related to the replacement
Reoperation within primary operative site		
4	1	Ankle gutter or heterotopic ossification debridement without exchange of metal components, with or without intact polyethylene exchange
6	2	Debridement of an osteolytic cyst without exchange of metal components, with or without intact polyethylene exchange
7	4	Deep infection or wound complication requiring operative debridement (without exchange or metal components in ankle replacement), with or without intact polyethylene exchange
9	2	Implant failure leading to revision of metal components due to aseptic loosening, component fracture, or malposition (no infection)
Amputation		
11	1	Amputation above the level of the ankle

Abbreviations: CROCS, Canadian Orthopaedic Foot and Ankle Surgery’s Coding System for Reoperations Following Total Ankle Replacement and Ankle Arthrodesis; TAR, total ankle replacement.

Seven patients met the definition for loosening of components^3,19,34^: 5 tibial sided and 2 who had subsidence of the talar component. Of the 5 patients with tibial loosening, 2 patients had loosening in the sagittal plane including the same patient who was originally malaligned in the coronal plane with progressive talar cysts that required subtalar fusion with bone grafting (CROCS 6). Two patients had loosening of the tibial component in the coronal plane, and 1 patient had loosening in both the coronal and sagittal planes, none of whom appeared to have any association with progressive cysts or reoperations. Two patients had loosening of the talar component: one who had subsidence in the posterior aspect of the talus with progressive cysts present, although clinically was asymptomatic (CROCS 1), and the other who had a change in the alignment of the talar component with cysts present on postoperative and most recent radiographs with an enlarging talar cyst. This patient was symptomatic and underwent subtalar fusion with bone grafting (CROCS 6) for concern of impending fracture. ICCs with 95% CIs were 0.72 (0.58, 0.83), 0.79 (0.68, 0.88), 0.96 (0.93, 0.98), 0.94 (0.90, 0.97), and 0.93 (0.87, 0.96) for the alpha, beta, gamma, and A and B measurements respectively.

### Implant Survivorship and Reoperations

**Figure 4.**
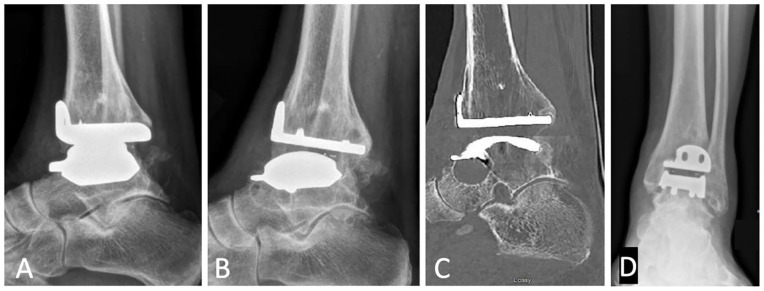
Imaging of patient with progressive subtalar cyst, (A) lateral radiograph postoperatively prior to cyst development, (B) lateral radiograph 6 years postoperatively, (C) sagittal computed tomography scan 6 years postoperatively, and (D) anteroposterior radiograph postoperatively with coronal malalignment.

To our knowledge, 39 patients (76.5%) have not required any subsequent operations after TAR and 12 patients (23.5%) required a reoperation (Table 5). Five patients categorized within CROCS 2, 3, 4, and 6 only required 1 subsequent operation, including a planned removal of syndesmotic fixation 4 months postoperatively after identifying a syndesmotic injury intra-operatively; a calcaneal osteotomy with opening wedge distal tibial osteotomy 4 months postoperatively for pain and increasing malalignment of the tibial component; medial gutter osteophyte debridement via arthroscopy 3.5 years postoperatively for anteromedial pain; subtalar fusion and talus cyst grafting 6 years postoperatively for impending fracture (Figure 4); and subtalar fusion with left proximal tibia bone graft harvesting, polyethylene component exchange, and debridement and bone grafting of talar cyst for fracture through the talar cyst.

Of importance, 2 (3.9%) patients required reoperation in the way of revision due to implant failure (CROCS 9). Indications included impending failure due to posterior ankle subluxation for one patient and chronic pain and osteolysis. These revisions were performed at 2 and 3 years postoperatively from the index operation; and these 2 patients have not required anymore subsequent procedures.

Four patients (7.8%) had deep infections (CROCS 7), 3 of which only required one subsequent operation at 2, 4 and 10 weeks postoperatively. One patient with deep infection required multiple operations ultimately requiring a pedicle muscle flap after eradicating the infection. A fifth patient (CROCS 11) required amputation above the level of the ankle; he had a previously infected ankle and arthroplasty was attempted as a salvage procedure which was unsuccessful.

## Discussion

This study reports a high prevalence of cysts found in patients who have undergone TAR using the HINTEGRA prosthesis. In total, 37 (73%) of 51 HINTEGRA prosthesis had cysts identified. Rates vary immensely in the literature from 0% to 86%,^13^ with this study supporting the higher end of the spectrum and substantially higher than other studies specifically evaluating the HINTEGRA prosthesis; 37.4%,^34^ 40%^6^ and 48%.^9^ Heterogeneity in methods and definitions of radiographic descriptions likely account for some variation as this study had an unbiased radiologist trained in musculoskeletal imaging examine radiographs. This may have improved detection sensitivity at the expense of clinical specificity. This likely led to identifying more cysts, some of which were noted to be present in the postoperative radiographs and may not represent cysts related to osteolysis.

Ligament imbalance and component malalignment can create high joint pressures and lead to osteolysis.^19^ Our results are supportive of this hypothesis, as the one patient who had tibial malaligned in the coronal plane on initial postoperative radiographs developed prosthesis loosening, fractured through an osteolytic talar cyst and required reoperation. It appears that malalignment in the sagittal plane is less threatening, as although 5 patients met the criteria for sagittal plane malalignment, none of them required any subsequent surgery. These results support recent literature with sagittal alignment having no relation to implant failure^10^ and surgeons advocating for custom sagittal cuts based on preoperative joint alignment,^33^ which could result in the tibial component meeting the definition for malalignment^19^ used in this study yet only benefit these patients.

Cyst prevalence increase throughout radiographic follow-up as 41 patients had at least one new cyst at their most recent radiographic follow-up. This has not been previously reported for the HINTEGRA prosthesis as Yoon et al^34^ (2014) identified a decreasing number of new cysts with time. One likely explanation for this is the length of radiographic follow-up was almost double in this study. The HINTEGRA prosthesis also has dual Ti-HA coating and a mobile-bearing, producing smaller, granular and more reactive particles with both which have been associated with increased cysts.^1^

Two types of osteolysis have been described: mechanical osteolysis, small non-progressive cysts with an early onset, attributed to stress shielding phenomena and biomechanical alterations at the bone-implant interface and ballooning/expansile osteolysis, late onset large progressive lesions due to a chemical phenomenon related to wear particles.^34^ A significant difference in the progression of osteolytic lesions based on whether the lesion was detected earlier or later than 1 year after TAR was found with half of the late-onset lesions showed continuous progression against only 9.5% in the early-onset group.^34^ This study supports this theory as 34 of the 43 (79%) cysts identified up to 1-year postoperative were less than 10 mm in diameter, and although 17 (39.5%) of these progressed to a larger size category, only 4 (9.3%) enlarged by 2 categories none of which required reoperation suggesting early identified cysts are clinically irrelevant and stable. Of the 41 new cysts identified after 1 year postoperation, 23 (56%) were larger than 10 mm and included the 2 patients who required reoperation for osteolysis. This suggests that large cysts identified in later follow-up and not present on prior radiographs are worrisome and should be followed closely.

This study showed excellent outcomes for the HINTEGRA prosthesis with a survivorship of 96.1% at 8.5 years. Other results have shown HINTEGRA to have a 10-year implant survival rates varying between 70% and 90%, which our results are in keeping with the literature.^2,14,22,36,37^ These results are equivalent to HINTEGRA prosthesis designer surgeons, who reported survival rates of 94% and 84% after 5 and ten years, respectively.^3^ Despite the excellent survival outcomes, the reoperation rate in this series was significant, with 23.5% patients requiring subsequent operations, including 2 who required implant revision. Reoperation rate in this series is comparable to other published series reporting on secondary reoperations for HINTEGRA prosthesis at 19% and 26.7%.^7,20,29,30,33^ It is important to note that 10 of the 12 only required one subsequent operation, however 4 (7.8%) patients had deep infections and required reoperation, which was slightly higher than literature describing a rate of 1.1-6.0%.^22,26,36^

Osteolysis has been reported as the most common reason for TAR revision,^11,16^ accounting for 28% to 38% of revisions^22,25^ with literature to suggest lower rates of implant loosening with the HINTEGRA prosthesis.^37^ In this study, one of the 2 patients (50%) requiring revision in this study were due to osteolysis and 2 other patients (CROCS 6) required reoperation due to osteolysis. It is important to note that osteolysis can occur without implant loosening and impending fracture is a reason for reoperation. Although this study contained a small population group, osteolysis accounts for 20% of unplanned reoperations.

Limitations of this study include the retrospective study design lacking patient demographic information, limited radiographic follow-up, and no patient-reported outcomes, although others report no substantial difference in clinical outcomes and pain between patient with and without osteolysis.^34^ Furthermore, additional procedures to obtain osseus and ligamentous balance were not included which could influence alignment and therefore the development of osteolysis. CT has been concluded as a more accurate method for detection and quantifying cysts^17^; however, plain radiographs remain the initial radiographic assessment because of its ease and cost. Analyzing only 1 time point of radiographs after 1 year also prohibited us from further delineating ballooning osteolysis, as some of these cysts are known to develop after 1 year postoperatively.^34^ This may have resulted in large cysts found on most recent radiographs being categorized as new cysts in this study. Finally, despite a high volume of TARs performed by 1 surgeon, the high presence of cysts with low number of failures and reoperations makes it difficult to power any statistical analysis regarding the radiographic formation, location, and progression of cysts and the relation between cysts and prosthesis alignment, reoperations, and implant survivorship.

## Conclusion

In conclusion, osteolysis is a very common finding after TAR using the HINTEGRA prosthesis, specifically on long-term radiographic follow-up. Most cysts do not appear clinically significant with no association with poor outcomes. However, progressive cysts, in particular ones not previously present at 1-year postoperatively as well as initial prosthesis coronal malalignment appear to be risk factors for the development of ballooning/expansile osteolysis, prosthesis loosening and reoperation. Early-onset cysts (<1 year) were often small and non-progressive, whereas late-onset or enlarging cysts were more frequently associated with adverse outcomes. This study also shows excellent and reliable outcomes of the HINTEGRA prosthesis comparable to designer surgeons with acceptable complication and revision rates. Larger studies are needed to better predict failure due to osteolysis and how we can prevent this from occurring.

## Supplemental Material

sj-pdf-1-fao-10.1177_24730114251363495 – Supplemental material for Osteolysis After HINTEGRA Total Ankle Replacement: Radiographic Patterns, Alignment Associations, and Long-Term Outcomes Supplemental material, sj-pdf-1-fao-10.1177_24730114251363495 for Osteolysis After HINTEGRA Total Ankle Replacement: Radiographic Patterns, Alignment Associations, and Long-Term Outcomes by Eric Locke, Roxane Heroux-Legault, Maram Alothman, Zaid Jibri, Brad Meulenkamp and Karl-André Lalonde in Foot & Ankle Orthopaedics
